# Post-therapy pathologic tumor volume predicts survival in gastric cancer patients who underwent neoadjuvant chemotherapy and gastrectomy

**DOI:** 10.1186/s12885-019-6012-7

**Published:** 2019-08-13

**Authors:** Xiaolong Tang, Qingsi He, Hui Qu, Guorui Sun, Jia Liu, Lei Gao, Jingbo Shi, Jianhong Ye, Yahang Liang

**Affiliations:** 1 Department of General Surgery, Qilu Hospital of Shandong University, No.107, West of Wenhua Street, Lixia District, Jinan, 250012 China; 2 Department of Health Management Center, Qilu Hospital of Shandong University, Jinan, 250012 China; 3 Qilu Medical College of Shandong University, Jinan, 250011 Shandong China

**Keywords:** Gastric cancer, Neoadjuvant chemotherapy, Tumor volume, Prognosis

## Abstract

**Background:**

To demonstrate that post-therapy pathological tumor volume (ypTV) should be considered as an independent prognostic factor in advanced gastric cancer (GC) patients who underwent neoadjuvant chemotherapy (NAC) and gastrectomy.

**Methods:**

A total of 253 GC patients who received gastrectomy between January 2010 and December 2016 in our hospital were enrolled in this study. Clinicopathologic factors were evaluated using univariable and multivariable analysis. ypTV was calculated using π* (tumor diameter/2)^2^ *tumor invasion depth (cm^3^).

**Results:**

Cut-point survival analysis demonstrated that the appropriate cut-offs for ypTV were 3, 6, 10, and 19 (cm^3^). Patients with tumor volumes of 0–3.0, 3.1–6.0, 6.1–10.0, 10.1–19.0, ≥19.1 cm^3^ were defined as ypTV1, 2, 3, 4a and 4b. Using multivariable analysis, the tumor volume (ypTV stage, *P* < 0.05), ypN stage (*P* < 0.05), response to NAC (*P* < 0.05), vascular invasion (*P* < 0.05) and ypTvNM staging (*P* < 0.05) were independent prognostic factors. Kaplan-Meier analysis demonstrated that the 8th AJCC/UICC ypTNM staging was not a significant predictor for survival (*P* > 0.05); however, our newly defined ypTvNM staging was a significant predictor for survival (*P* < 0.05).

**Conclusions:**

ypTV should be considered as an independent prognostic factor for GC patients after NAC. ypTvNM staging should be recommended to improve the accuracy of prognostic prediction for GC patients who received NAC plus gastrectomy.

## Background

Due to the poor prognosis of patients with advanced gastric cancer (GC), neoadjuvant chemotherapy (NAC) has been used to improve survival [1]. There have been several NAC regimens that have been suggested by the National Comprehensive Cancer Network (NCCN) guidelines version 1.2017. These include combinations of fluorouracil and cisplatin, fluoropyrimidine and oxaliplatin, and ECF (epirubicin, cisplatin, and fluorouracil) [2]. To determine the most appropriate combination, a staging system which could reflect accurately the overall survival is essential. Shrinkage of tumors and surrounding lymph nodes after neoadjuvant chemotherapy complicates post-surgical pathological classification. However, prognostic factors are ambiguous for patients who had received NAC and gastrectomy.

The tumor-node-metastasis (TNM) classification for gastric cancer has been considered as the best classification system since it provides prognostic estimation and guidance for patients. However, several studies have suggested that traditional TNM classification for GC may not be the most optimal [3]. For several types of malignant tumors, tumor volume (TV) is an important prognostic factor [4]. However, only a few reports have evaluated the relationship between TV and prognosis for advanced GC patients who received NAC. This study was designed to assess the potential impact of tumor volume on long-term survival of patients treated with neoadjuvant chemotherapy and gastrectomy for cancer.

## Methods

### Patients

We retrospectively analyzed 253 GC patients who received gastrectomy at Shandong University Qilu Hospital, during January 2010 and December 2016. All patients were diagnosed with gastric adenocarcinoma by histopathological examination and received neoadjuvant chemotherapy before surgery. Endoscopic ultrasonography (EUS) was used to evaluate pT stage prior to surgery. Clinical stage pre- and post-surgery were evaluated for all patients using enhanced computed tomography (CT) and/or magnetic resonance imaging (MRI). All patients that were included have potentially curable disease at the onset of staging. However, a total of 26 patients were found to have distant metastasis (IV stage) during laparotomy and received palliative surgery. These patients had peritoneum metastasis (18 cases), ovarian metastasis (5 cases) and liver metastasis (3 cases). Other 227 patients without distant metastasis (I-III stage) received radical gastrectomy and D2 lymphadenectomy. Patients with distant metastasis (IV stage) were found intraoperation and received palliative gastrectomy. The decision to perform total or subtotal gastrectomy was primarily based on the location and diameter of the tumors. To eliminate confounding factors, patients who received synchronous chemoradiotherapy before surgery were excluded. Nasogastric tube placement was not routinely performed. Intravenous or epidural anesthesia was used for post-surgical pain management. Patients were initiated on an oral diet on the 3th or 4th day after surgery. Patients without complications or other medical problems were discharged between 7 and 11 days after surgery.

### Pathological analysis

Clinicopathological data, including gender, age, location of tumor, histological differentiations, ypT, ypN, ypTNM, distant metastasis, surgical type, and response rate were evaluated. Tumor volume was defined as follows: ypTV was defined as π*(tumor diameter/2)^2^*tumor invasion depth (cm^3^). The tumor diameter was defined as the maximum diameter of the tumor. TV was calculated by two pathologists. Tumors of Borrmann’s type IV was defined as ypTV4b. To evaluate the prognostic value of ypTV, we included ypTV to the ypTNM staging criteria and defined the tumor volume- node- metastasis (ypTvNM) staging system. Histological differentiation was classified as well- differentiated, moderately- differentiated, poorly- differentiated, and signet- ring cell carcinoma. Histological types were classified based on the Lauren classification. Tumor location was classified as proximal, middle or distal. For quality control, the number of metastatic lymph nodes were evaluated by two independent pathologists. All histopathologic data were collected and determined using the 8th edition American Joint Committee and Union International Center Cancer (AJCC/UICC) TNM classification. Tumor response after neoadjuvant chemotherapy was determined using the Response Evaluation Criteria in Solid Tumors (RECIST), version 1.1.

### Follow-up

After surgery, all patients were managed using standardized follow-up protocols. All patients were regularly followed-up for at least 3 years post-surgery. Follow up investigations were scheduled at 3-month intervals for the first 2 years, at 6-month intervals for up to 5 years, and then every year thereafter. The median follow-up period was 67 (range: 22–116) months, and the last follow-up date was November 15, 2018. The OS rate was calculated from the date of surgery until the final follow-up date or death.

### Statistical analysis

We used the SAS 9.4 (SAS Institute Inc., Kerry, USA) software for all statistical analysis. Univariable and multivariable analyses were used to identify the most significant classification that correlated with prognosis. Based on ypTV, we used the Life Tables method to analyze OS rates. χ^2^ or Fisher’s exact test was used to assess the relationship between ypTV and clinicopathological factors. Overall survival curves were constructed using the Kaplan-Meier method based on the date of surgery to the final follow-up or death. The log-rank test was used to assess statistical differences between the survival curves. Cut-point survival analysis was used to determine the optimal cut-offs for ypTV. All parameters that were *P* < 0.05 in univariable analysis were included for multivariable analysis. Independent prognostic factors were identified using the Cox proportional hazards regression model. Statistical significance was set as *P* value < 0.05.

## Results

### Clinicopathological outcomes

Of the 227 patients who received radical gastrectomy, 119 (52.4%) patients were male and 108 (47.6%) were female, with a median age of 58 years (range, 25–75 years). The mean body mass index (BMI) was 24.1 ± 2.4 kg/m^2^. Tumors were located in the lower third of the stomach in 52 (22.9%) patients, in the middle third of the stomach in 102 (44.9%) patients, in the upper third of the stomach in 42 (18.5%) patients, and in the whole stomach in 31 (13.7%) patients. All patients enrolled in this study underwent gastrectomy with lymphadenectomy. A total of 118 (51.9%) patients underwent subtotal gastrectomy, 68 (30.0%) patients underwent total gastrectomy, and 41 (18.1%) underwent combined organ resection. A median of 28 (15–78) lymph nodes per patient was dissected for histopathological examination post-surgery. None of the patients died during hospitalization.

Neoadjuvant chemotherapy was administered based on the NCCN guidelines according to the tumor stage, physical condition and patient willingness. If we suspected that the tumor has invaded into adjacent tissues or organs, or metastatic lymph nodes beyond the region of D2 lymphadenectomy, neoadjuvant chemotherapy was considered. 5-fluorouracil, leucovorin and oxaliplatin was used for NAC before gastrectomy. Most of the patients received fluorouracil (capecitabine) and cisplatin/oxaliplatin, while some received ECF, DCF, paclitaxel, and carboplatin. Patients usually received 3 to 4 cycles of neoadjuvant chemotherapy. This was determined by tumor response and patient’s tolerance. Based on the RECIST criteria, 170 patients were evaluated as partial response (PR), 35 patients with stable disease (SD) and 22 patients with progressive disease (PD) after neoadjuvant chemotherapy. None of the patients had complete response (CR) after post-surgical pathological diagnosis. After surgery, all patients received 6–8 courses of adjuvant chemotherapy.

### Univariable survival analysis

**Fig. 1:**
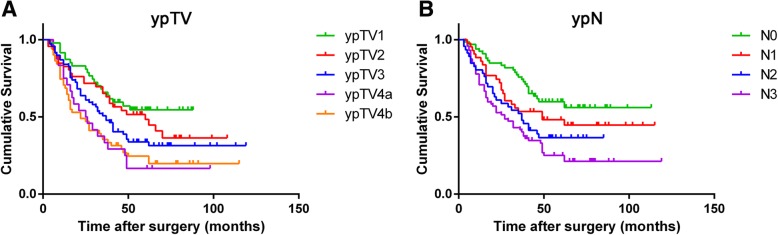
Survival curves of patients based on ypTV and ypN **a**. ypTV was identified as significantly correlated with prognosis (*P* < 0.001). **b**. ypN was identified as significantly correlated with prognosis (*P* < 0.001)

Cut-point survival analysis demonstrated that the appropriate cut-offs for ypTV were 3, 6, 10, and 19 (cm^3^). Under this classification, ypTV subgroups were determined to have statistically significant survival differences. In contrast to pT staging system, patients with tumor volumes of 0–3.0, 3.1–6.0, 6.1–10.0, 10.1–19.0, ≥19.1 cm^3^ were defined as ypTV1, 2, 3, 4a and 4b. Based on this criteria, 227 patients were reclassified as follows: 51 (20.2%) patients were ypTV1, 49 (19.4%) patients were ypTV2, 73 (32.2%) patients were ypTV3, 23 (10.1%) patients were ypTV4a and 31 (13.7%) patients were ypTV4b. Using Kaplan-Meier analysis, response to neoadjuvant chemotherapy (*P* < 0.001), surgical type (*P* = 0.009), vascular invasion (*P* = 0.028), Borrman’s classification (*P* = 0.018), ypT stage (*P* < 0.001), ypTV (*P* < 0.001), ypN stage (*P* < 0.001) and ypTvNM stage (*P* < 0.001) were identified as significantly correlated with prognosis (Table 1). Figure 1a,b shows the patient survival curves based on ypTV and ypN (All *P* < 0.001).

**Table 1:** Univariable Survival Analysis

Parameters	No. of patients (*n* = 227)	Median survival (months)	χ^2^	*P* ^*^
Gender			1.73	0.179
Male	119	48 (21–78)		
Female	108	43 (19–65)		
Age (years)			1.22	0.270
≤ 60	132	38 (16–55)		
> 60	95	41 (20–62)		
Response to neoadjuvant chemotherapy			19.40	< 0.001
PR	170	62 (22–81)		
SD	35	35 (16–48)		
PD	22	25 (13–41)		
Location of tumor			0.76	0.859
Upper	42	41 (22–49)		
Middle	102	37 (20–51)		
Lower	52	49 (16–72)		
Diffuse infiltration	31	38 (16–57)		
Differentiation of tumor			8.77	0.591
High	80	35 (16–71)		
Middle	46	39 (33–51)		
Poor	49	33 (19–57)		
Signet ring cell	52	31 (21–49)		
Surgical type			9.45	0.009
Subtotal	118	50 (21–79)		
Total	68	31 (18–62)		
Combination of organs	41	34 (19–56)		
Vascular invasion			4.82	0.028
No	161	44 (20–75)		
Yes	66	32 (12–43)		
Borrman’s classification			10.13	0.018
1	32	43 (14–68)		
2	56	63 (30–84)		
3	97	40 (18–64)		
4	41	28 (20–45)		
Lauren’s classification			1.28	0.526
1	100	46 (20–69)		
2	82	40 (16–71)		
3	43	29 (16–51)		
ypT stage			35.95	< 0.001
ypT1	12	58 (19–81)		
ypT2	32	47 (16–65)		
ypT3	63	62 (29–91)		
ypT4a	98	27 (16–47)		
ypT4b	22	22 (14–38)		
Tumor volume (ypTV)			25.74	< 0.001
ypTV1	51	53 (27–78)		
ypTV2	49	47 (24–76)		
ypTV3	73	41 (22–64)		
ypTV4a	23	31 (14–49)		
ypTV4b	31	17 (10–47)		
Number of resected lymph nodes			1.77	0.183
≤ 16	41	38 (18–54)		
> 16	186	41 (19–69)		
ypN stage			18.27	< 0.001
ypN0	66	48 (37–61)		
ypN1	50	40 (16–70)		
ypN2	39	35 (20–59)		
ypN3	72	28 (15–50)		
ypTNM stage			2.40	0.210
I	31	57 (41–82)		
II	48	47 (36–70)		
III	148	32 (15–49)		
ypTvNM stage			21.23	< 0.001
I	37	67 (41–89)		
II	57	44 (22–69)		
III	133	32 (14–62)		

*Log rank test

### Multivariable survival analysis

All parameters with *P* value < 0.05 in the univariable survival analysis were enrolled in the multivariable survival analysis. Using multivariable analysis, the tumor volume (ypTV stage, *P* < 0.05), ypN stage (*P* < 0.05), ypTvNM stage (*P* < 0.05), response to neoadjuvant chemotherapy (*P* < 0.05) and vascular invasion (*P* = 0.004) were confirmed to be independent prognostic factors. The 5-year survival rate for patients with different ypTV classifications were stratified by tumor volume. As shown in Table 2, patients with different ypTV classifications had significant differences in survival (*P* = 0.045, 0.029, 0.021, and 0.006, respectively).

**Table 2:** Multivariable Survival Analysis

Parameters	B	SE	HR	Hazard ratio (95% CI)	*P*
Tumor volume (ypTV)					
ypTV1	–	–	–	–	–
ypTV2	0.10	0.30	1.08	1.01–1.61	0.045
ypTV3	0.43	0.21	1.53	1.02–2.31	0.042
ypTV4a	0.69	0.32	1.99	1.06–3.72	0.032
ypTV4b	0.89	0.30	2.44	1.35–4.41	0.003
ypN stage					
N0	–	–	–	–	–
N1	0.63	0.27	1.88	1.11–3.2	0.019
N2	0.65	0.22	1.92	1.25–2.93	0.003
N3	0.70	0.27	2.01	1.18–3.42	0.010
ypTvNM stage					
I			1.00		
II	0.22	0.07	1.25	1.09–1.43	0.001
III	0.64	0.23	1.90	1.22–2.96	0.005
Response to neoadjuvant chemotherapy					
PR	–	–	–	–	–
SD	0.44	0.23	1.65	1.08–2.43	0.041
PD	0.61	0.23	1.85	1.17–2.92	0.009
Vascular invasion					
No	–	–	–	–	–
Yes	0.45	0.22	1.58	1.03–2.4	0.035

**Fig. 2:**
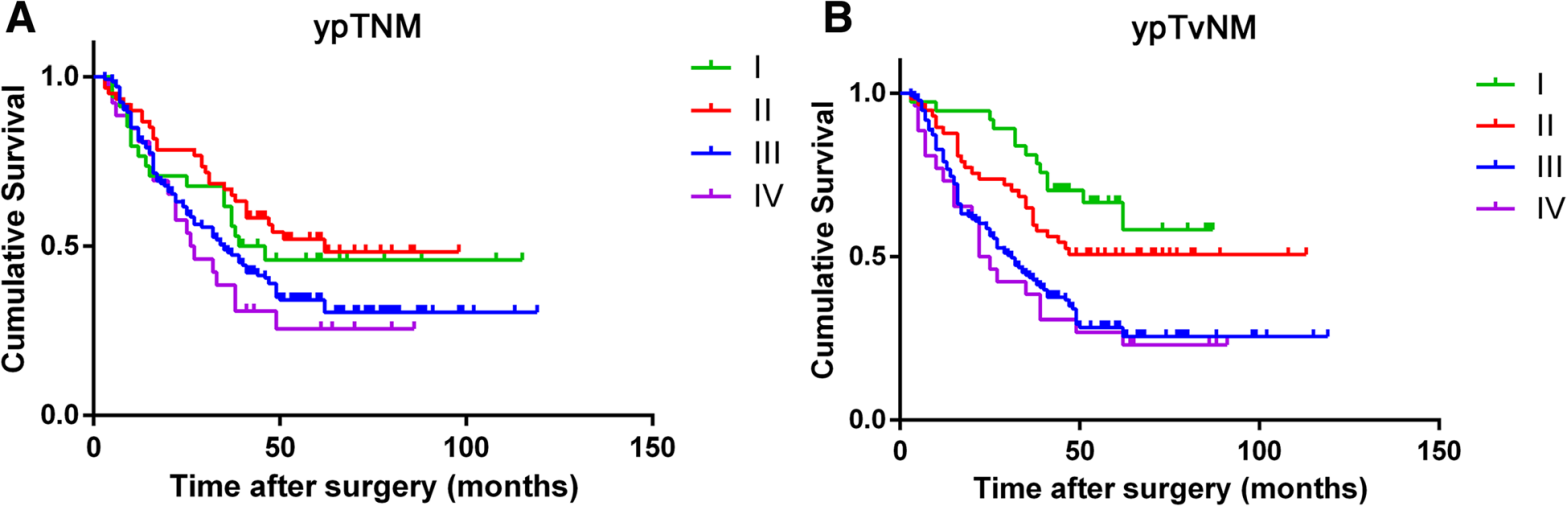
Patient survival curves based on the 8th AJCC TNM staging system and our suggested ypT_V_NM categorization system **a**. Patients with ypTNM stage I, II and III did not show significant differences in survival (*P* = 0.476, 0.360, respectively). Only ypTNM stage IV demonstrated significant worse survival (*P* < 0.001). **b**. Significant differences in survival between patients with ypTvNM stage I, II, III and IV (*P* = 0.036, 0.001, < 0.001, respectively; Fig. 2b)

The 8th AJCC TNM staging system with our suggested ypTvNM categorization system was then directly compared. The 26 patients with distant metastasis (stage IV) were included in the ypTNM and ypTvNM staging systems. Detailed survival differences are shown in Table 3. Patients with ypTNM stage I, II and III did not show significant differences in survival (*P* = 0.476, 0.360, respectively). Only patients with distant metastasis (ypTNM stage IV) showed significantly worse survival (*P* < 0.001; Fig. 2a). However, there were significant differences in survival between patients with ypTvNM stage I, II, III and IV (*P* = 0.036, 0.001, < 0.001, respectively; Fig. 2b).

**Table 3:** Detailed survival differences between the 8th AJCC TNM staging system and the ypT_V_NM categorization system

The 8th AJCC ypTNM stage (*n* = 253)				ypTvNM stage (*n* = 253)			
ypTNM	No. of patients	Median survival (months)	*P*	ypTvNM	No. of patients	Median survival (months)	*P*
I	31	57 (41–82)	–	I	37	67 (41–89)	–
II	48	47 (36–70)	0.476	II	57	44 (22–69)	0.036
III	148	32 (15–49)	0.360	III	133	32 (14–62)	0.001
IV	26	17 (5–22)	< 0.001	IV	26	17 (5–22)	< 0.001

## Discussion

Neoadjuvant chemotherapy has become increasingly popular to treat potentially resectable advanced GC. However, evaluating prognosis after neoadjuvant chemotherapy has become problematic [5]. Due to shrinkage of tumors and surrounding lymph nodes after neoadjuvant chemotherapy, indicators that are used to reflect OS for GC patients remains controversial [6]. In 2017, the 8th edition of the AJCC/ UICC TNM staging system was the first to introduce the ypTNM staging system for GC patients who received neoadjuvant chemotherapy [7]. However, the ypTNM staging system was based on only 700 cases and was similar to the pathologic TNM classification for GC without neoadjuvant chemotherapy. We conducted the current study to investigate a more accurate ypTNM staging system for GC patients who received neoadjuvant chemotherapy.

Tumor volume (TV) has been demonstrated as an important prognostic factor for several malignant tumor types. Hollmann et al. found that TV was an effective method to evaluate response to chemotherapy and predict prognosis in patients with prostate cancer [8]. Jiang et al. evaluated TV in GC patients without NAC. They reported that TV was significantly associated with prognosis, and pTV staging could be more reliable compared to UICC/AJCC on cancer pT system for prognostic assessment [9]. Takenaka et al. reported the prognostic impact of TV in patients with clinical stage IA non-small cell lung cancer [10]. The calculation of TV in these publications were mostly based on CT/ MRI images using specialized software. However, our method of calculating TV required to two measurements: the tumor diameter and tumor invasion depth. This provided a much simpler method for surgeons to assess tumor burden and prognose GC patients after NAC.

Based on our data, patients who underwent NAC and gastrectomy could be reclassified into five groups according to the ypTV classifications (*P* < 0.05). Cut-point survival analysis showed that the most appropriate cut-offs for ypTV were 3, 6, 10, and 19 cm^3^. Several studies have shown that the ypT classification system does not completely reflect the tumor burden [11]. However, ypTV was linearly correlated with tumor burden and was the best indicator. Larger tumor burden generally indicated a longer duration and/or higher proliferation of tumor growth, with higher possibility of lymphatic metastasis, and potential distant metastasis [12]. In multivariable analysis, the ypTV classification was an independent prognostic factor (*P* < 0.05), while the ypT classification was not (*P* > 0.05). These results indicated that the ypTV classification may be superior to the ypT classification in our cohort. Inclusion of ypTV improved the accuracy of tumor staging for patients with advanced GC after NAC and gastrectomy.

ypTV was incorporated into TNM staging for patients with advanced GC who underwent NAC and gastrectomy. We found that the ypTvNM classification was the most appropriate prognostic classification for predicting overall survival (OS) (*P* < 0.001) versus that of the 8th edition AJCC/ UICC ypTNM classification. The results of our study indicated that inclusion of ypTV into the new ypTvNM classification for patients with advanced GC would help enable a more exact prediction of prognosis. In this study, we try to provide a new tool for both pathologists and surgeon to evaluate the prognosis of GC patients who received NAC. TV could be easily calculated using post-surgical pathology reports and will not bring extra work for the pathologists.

Although all the patients included in this study received CT or/and MRI scans, a total of 26 patients were found to have distant metastasis (IV stage) during laparotomy. The decision for palliative resection was made when tumors were found to be unresectable in patients scheduled for potentially curative gastrectomy. Unresectable tumors were associated with significant perioperative morbidity and mortality as well as limited OS. In fact, the rate of curative resections was significantly increased with NAC for advanced GC patients [13].

There were several limitations of our study. The patient cohort in this study was relatively small and there was a lack of standardized NAC regimens which may have affected patient survival [14]. Prognosis evaluation for patients with advanced GC after NAC remains an issue. Future studies using larger patient cohorts with longer follow-up periods are required to validate our findings.

## Conclusions

ypTV may be a potential independent prognostic factor for patients with advanced GC who had undergone NAC. Incorporating ypTV into the TNM staging may compensate for the limitations of the ypT classification. The ypTvNM classification could be recommended as a more accurate staging for GC patients who had undergone NAC and gastrectomy.

## Data Availability

The datasets used and/or analyzed in this study are available from the corresponding author on reasonable request.

## Abbreviations

AJCC/UICC: American Joint Committee and Union International Center Cancer
GC: gastric cancer
NAC: neoadjuvant chemotherapy
NCCN: National Comprehensive Cancer Network
RECIST: Response Evaluation Criteria in Solid Tumors
TNM: tumor-node-metastasis
TV: tumor volume
TvNM: tumor volume-node-metastasis
